# High radiation dose in chemoradiotherapy followed by immunotherapy with durvalumab in patients with stage III non-small cell lung cancer does not increase risk for pneumonitis

**DOI:** 10.1007/s00066-025-02369-0

**Published:** 2025-02-13

**Authors:** Felix Schragel, Melanie Matousek, Christoph Resl, Gudrun Kreye, Nguyen-Son Le, Peter Errhalt, Petra Georg, Klaus Hackner

**Affiliations:** 1 Division of Pneumology, University Hospital Krems, Karl Landsteiner University of Health Sciences, Krems, Austria; 2 Division of Radiotherapy-Radiation Oncology, University Hospital Krems, Karl Landsteiner University of Health Sciences, Krems, Austria; 3 Division of Internal Medicine II, University Hospital Krems, Karl Landsteiner University of Health Sciences, Krems, Austria

**Keywords:** Pneumonitis, Lung cancer, Immunotherapy, Chemoradiotherapy, PACIFIC

## Abstract

**Purpose:**

Consolidation immunotherapy with immune checkpoint Inhibitor (ICI) Durvalumab is an effective treatment for inoperable stage III non-small cell lung cancer (NSCLC) patients with a PD-L1 expression ≥ 1% after definitive curative concurrent chemoradiotherapy (CCRT). While this approach is widely used as standard therapy, it carries an increased risk of immune-related and radiation-induced pneumonitis. Currently, there is no data on pneumonitis in *patients receiving CCRT with an overall dose of 70* *Gy (Gy) compared with the standard* protocol of 60 Gy ± 10% in this setting.

**Methods:**

This study analyzed retrospective data from 39 patients with unresectable NSCLC treated with CCRT. Patients received either 70 Gy (*n* = 29) or lower than 70 Gy total dose (*n* = 10) in 2 Gy fractions. Cases of pneumonitis were further classified as RI‑P (Radio-induced Pneumonitis) and ICI‑P (ICI Pneumonitis) based on clinical and radiological findings.

**Results:**

Of the 39 patients, 15 (38.5%) developed pneumonitis, with 10 out of 29 (34.5%) in the 70 Gy group and five out 10 (50%) in the < 70 Gy group. There was no significant difference in pneumonitis and in occurrence of ICI‑P vs. RI‑P (26.7% vs. 73.3%) within both groups. The 70 Gy group showed a significant benefit in mortality (*p* = < 0.001). Overall survival (OS) differed significantly between groups (*p* =0.028).

**Conclusions:**

70 Gy radiation dose for CCRT followed by durvalumab is a safe regimen and may provide clinical benefits in NSCLC patients compared to lower doses. Pneumonitis incidence aligns with previous literature. The higher dose is associated with improved overall survival, and reduced disease progression, potentially due to a longer consolidation time.

## Introduction

Immune-checkpoint inhibitor (ICI) treatment after definitive concurrent chemoradiotherapy (CCRT) represents a standard regimen for unresectable stage III non-small lung cancer in patients with a programmed death-ligand 1 (PD-L1) expression ≥ 1%. Antonia et al. (PACIFIC Trial; 2017) showed that patients receiving Durvalumab as consolidation after CCRT had a significantly increased overall survival compared to the control group without ICI [1]. However, previous trials and reviews have noted that the use of ICI after radiotherapy may lead to a higher incidence of immune-checkpoint inhibitor induced pneumonitis (ICI-P). Furthermore, not only ICI can cause adverse events, but radiation therapy alone or in combination with chemotherapy can also be considered as a relevant risk factor for radiotherapy-induced pneumonitis (RI-P) [1–5].

In the PACIFIC trial the overall dose applied was 60 Gy ± 10%. This protocol was based on available evidence from several studies: a trial by Bradley et al. showed that 74 Gy radiation given in 2 Gy fractions during CCRT was actually not superior to 60 Gy in patients with stage III NSCLC, and maybe was even more harmful to surrounding structures [6]. ‘Lower’ dose radiation was also considered as a favourable treatment option by other authors [3–5]. Schild et al. highlight that doses of radiotherapy above 60 Gy were associated with more grade 3 and 4 adverse events than radiotherapy with 60 Gy. In addition, grade 5 adverse events and even deaths associated with radiotherapy were more common with doses above 60 Gy [3]. Therefore, a total radiation dose of 60 Gy ± 10% (54 Gy to 66 Gy) and a mean lung dose (MLD) of < 20 Gy and/or V20 (total lung volume receiving 20 Gy) of < 35% is often used as a dose constraint in the current literature, including the PACIFIC trial [1, 3–7]. On the other side, Brower et al. analyzed > 30,000 patients with stage III NSCLC who underwent CCRT and found that a dose escalation above 60 Gy was associated with improved overall survival (OS) [8]. However, a survival plateau was found above radiation doses > 70 Gy. Therefore, it is suggested that dose escalation should be limited to this range [8].

Selected patients at the study site (University Hospital Krems) were treated with total doses of 70 Gy during CCRT, which may lead to a higher incidence of pneumonitis, especially with a following ICI treatment with Durvalumab. However, some patients received less than 70 Gy due to larger tumor size, as the overall radiation volume would exceed the limits. These patients served as the control group.

The aim of this study was to evaluate the actual overall incidence of pneumonitis in this setting and to investigate if it correlates with radiation dose. Furthermore, the analysis aimed to show, if there is correlation between radiation dose and the incidence of pneumonitis (both, radiation- and ICI-induced pneumonitis) in patients who received a total dose of 70 Gy followed by Durvalumab. The secondary objective was to compare the results with the current literature, in particular the PACIFIC trial, regarding pneumonitis cases. Further secondary endpoints were progression free survival (PFS) and OS.

## Materials and methods

### Study population

This retrospective study was conducted at the University Hospital Krems. The medical data of the patients was provided by the study site. Systemic treatment (i.e. Durvalumab after CCRT) was performed at various hospitals within Lower Austria according to the specification and decision of the study center, whereas diagnosis and CCRT only took place at the study site. Eligible patients were diagnosed with histologically or cytologically confirmed Union of International Cancer Control (UICC; 8th edition) stage III NSCLC, which was unresectable according to a multidisciplinary discussion. Staging was completed with FDG-PET-CT Scan and cranial MRI prior to irradiation. All patients were treated between 2017 and 2023 with CCRT with a minimum total dose of 60 Gy and a maximum total dose of 70 Gy followed by ICI therapy with Durvalumab. Durvalumab therapy has been started within a mean of 14 (6–20) days after CCRT and was administered intravenously at a dose of 10 mg/kg every two weeks for a duration of one year to patients with no disease progression after two or more cycles of CCRT. Patients who have not experienced any adverse events after six cycles were then switched to a four week cycle with a double dose. The administration of Durvalumab was continued until tumor progression, appearance of an adverse event or withdrawal of consent.

The estimated mean drug doses and V20 values were utilized as essential criteria for total radiation doses. Patients with high MLD, which imposed a high dose constraint, were administered a total prescription dose less than 70 Gy. To minimize the risk of symptomatic pneumonitis to less than 20%, specific limits were established, such as the MLD should be equal to or less than 20 Gy, and the V20 limit for both lungs should be equal to or less than 40%. If any of these criteria were exceeded, the total prescribed dose was reduced accordingly. Chemotherapy was administered with Carboplatin or Cisplatin in combination with Navelbine. Durvalumab was started within 21 days after CCRT was finished, with a 2- or 4‑weeks interval, and was administered for a maximum of 12 months.

In accordance with the results of Chun et al. (RTOG 0617) [9], which showed that new techniques reduce lung morbidity compared to 3D conformal radiotherapy, all patients were treated with volumetric intensity modulated arc therapy (VMAT). As both patient groups were treated with the same planning technique it should not affect treatment outcomes or toxicity.

The institutional planning procedure was as followed:

Target volume concept included the following: A 4D-CT was used for treatment planning. The Internal Clinical Target Volume (iCTV) for primary tumor and pathological lymph nodes was the Internal Gross Target Volume (iGTV) + 7 mm respecting anatomical boundaries. In case of pathologic lymph nodes, the whole lymph node level was delineated into the Clinical Target Volume (CTV). No elective lymph node radiation was performed. The planning target volume (PTV) was the CTV + 5 mm.

Dose constraints for lung are shown in Table 1.

**Table 1:** Dose constraints for lung

Mean dose	< 17 Gy (optimal)
	< 20 Gy (mandatory)
V40 Gy	< 10% (optimal)
V30 Gy	< 15% (optimal)
V20 Gy	< 20% (optimal)
	< 30% (mandatory)
V10 Gy	< 40% (optimal)
V5 Gy	< 50% (optimal)

### Endpoints

The evaluated endpoints included pneumonitis-free time, PFS and OS. Pneumonitis-free time was defined as the period from end of CCRT until the onset of a pneumonitis of any cause. PFS was defined as the time elapsed from the initiation of CCRT until the occurrence of disease progression. OS was calculated starting after the last day of CCRT using the Kaplan-Meier estimator method. Date of last follow-up was identified as either the date of death, last in-person visit or last research phone call.

### Pneumonitis classification

**Fig. 1:**
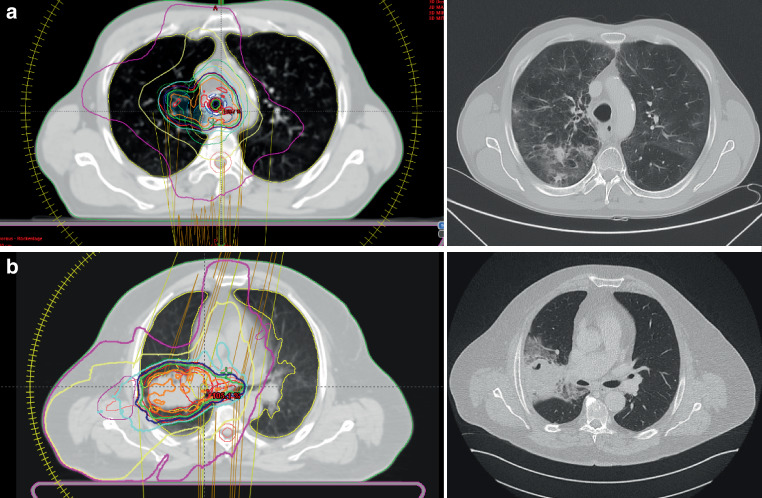
**a** Example of Immune-checkpoint inhibitor pneumonitis (ICI-P): Radiation-planning CT (left) and CT for pneumonitis evaluation (right). Bilateral Ground glass opacities with diffuse distribution and no sharp borders suggesting ICI-Pneumonitis. **b** Example of Radiation-induced pneumonitis: Radiation-planning CT (left) and CT for pneumonitis evaluation (right). Consolidation with sharp borders, unilateral and within the main areas of the radiation field

Pneumonitis was diagnosed based on clinical and radiological findings by a team of physicians, including an oncologist, a pulmonologist and a radiation oncologist. Other potential causes such as active infection or tumor progression, were ruled out first [7, 10–14]. Details on differentiation criteria are given in Table 2 and Fig. 1. Pneumonitis was graded according to the Common Terminology Criteria for Adverse Events (CTCAE) version 5.0 (Table 3).

**Table 2:** Pneumonitis type differentiation criteria. Modified from Fitzpatrick et al. [14]

RI‑P (Radiation-induced pneumonitis)	ICI‑P (Immune-checkpoint inhibitor induced pneumonitis)
Small areas within the radiation field	Larger areas
Unilateral	Often bilateral
Sharp borders	No sharp boards

**Table 3:** Common Terminology Criteria for Adverse Events (CTCAE) for Pneumonitis version 5.0

CTCAE Term	Grade 1	Grade 2	Grade 3	Grade 4	Grade 5
Pneumonitis	Asymptomatic; clinical or diagnostic observations only; intervention not indicated	Symptomatic; medical intervention indicated; limiting instrumental ADL	Severe symptoms; limiting self care ADL; oxygen indicated	Life-threatening respiratory compromise; urgent intervention indicated (e.g., tracheotomy or intubation)	Death

### Statistics

The statistical analysis was performed using SPSS Statistics 29.0.0.0 (IBM Corp., Armonk, NV, USA) and GraphPad Prism 6 (GraphPad Software, San Diego, CA, USA). Normal distribution was assessed using the Kolmogorov-Smirnov- und Shapiro-Wilk-tests. The significance level for all calculations was set at *p* < 0.05, and all tests were two sided.

Patients baseline and treatment characteristics were presented as either number for dichotomously pronounced variables (e.g. the prevalence of pneumonitis) or mean and median for continuous variables. To compare different treatment groups or evaluate the association between pneumonitis and these characteristics, categorical variables were analyzed using Fisher’s exact test or Chi-Square test, while continuous variables were analyzed using independent sample t‑tests or Mann-Whitney‑U tests depending on their normal distribution.

The overall survival time (months) was calculated from the end of CCRT until the last follow-up visit or death. Progression-free survival time (months) was calculated from the start of CCRT until the last follow-up visit or the timepoint of tumor progression. Pneumonitis-free survival time (months) was calculated from the end of CCRT until the last follow-up visit or the day of pneumonitis occurrence. All survival analysis were conducted using Kaplan-Meier curves, which estimated the probability of survival over time. Between group comparisons were performed using the log-rank test.

## Results

### Patients characteristics and incidence of pneumonitis

Table 4 offers an overview of the patients’ characteristics, with comparison of the 70 Gy (*n* = 29) and < 70 Gy (*n* = 10) total dose groups. There was no significant difference in age, gender, smoking status, disease stage, histologic type of NSCLC, lung function, performance status, size of the primary lesion or tumor location. Lung function testing was performed prior to histological sampling but was not routinely monitored after the completion of chemoradiotherapy.

**Table 4:** Overview of the study population

*N*	All patients	70 Gy overall doses	< 70 Gy overall doses	*p*-value
	39	29	10	
Age (years; median, IQR)	63 (59–67)	64 (59.5–67.5)	62.5 (56.7–67.2)	n. s.*
< 60a	10	7	3	–
*≥* *60a*	29	22	7	
**Gender, *n* (%)**				
Female	16 (41)	11 (37.9)	5 (50)	n. s.*
Male	23 (59)	18 (62.1)	5 (50)	–
**Smoking Status, *n* (%)**				
Never	2 (5.1)	1 (3.4)	1 (10)	n. s.*
Former + current	37 (94.9)	28 (96.6)	9 (90)	–
**Disease stage (UICC 8th edition), *n* (%)**				
IIIA	24 (61.5)	19 (65.5)	5 (50)	n. s.*
IIIB	13 (33.3)	8 (27.5)	5 (50)	–
IIIC	2 (5.1)	2 (6.9)	–	
**Histologic type, *n* (%)**				
SCC	15 (38.4)	13 (44.8)	2 (20)	n. s.*
Adenocarcinoma	23 (59)	16 (55.2)	7 (70)	–
Others	1 (2.6)	–	1 (10)	
**Tumor size primary lesion (mm), mean (SD)**				
–	37.0 (17.0)	36.5 (16.8)	38.5 (18.4)	n. s.*
**Performance status (ECOG), *n* (%)**				
0	17 (43.6)	15 (51.7)	2 (20)	n. s.*
1	21 (53.8)	13 (44.8)	8 (80)	–
2	1 (2.6)	1 (3.4)	–	
**Lung function (% predicted), mean (SD)**				
FEV1	74 (20.2)	73 (17.2)	79 (28.0)	n. s.*
FVC	94 (18.2)	92 (19.4)	99 (13.8)	–
FEV1%/FVC	62 (10.8)	62 (7.6)	61 (17.6)	
TLC	104 (19.3)	104 (15.5)	105 (28.8)	
DLCO/VA	75 (18.9)	74 (19.5)	79 (17.3)	
**Tumor location, *n* (%)**				
Upper lobe	21 (53.8)	16 (55.2)	5 (50)	n. s.*
Lower lobe	6 (15.4)	2 (6.9)	4 (40)	–
Central	15 (30.8)	11 (37.9)	1 (10)	
**Pneumonitis, *n* (%)**				
Yes	15 (38.5)	10 (34.5)	5 (50)	n. s.*
No	24 (61.5)	19 (65.5)	5 (50)	–
**Type of pneumonitis, *n* (%)**				
ICI‑P	4 (26.67)	3 (30)	1 (20)	n. s.*
RI‑P	11 (73.33)	7 (70)	4 (80)	–
**Pneumonitis CTCAE grade, *n* (%)**				
2	14 (93.33)	10 (100)	4 (80)	n. s.*
3	1 (6.67)	–	1 (20)	–
**Timing of Pneumonitis, *n* (%)**				
Acute toxicity (≤ 90 days)	6 (40)	4 (40)	2 (40)	n. s.*
ICI‑P	–	–	–	–
RI‑P	6 (100)	4 (100)	2 (100)	
Late toxicity (> 90 days)	9 (60)	6 (60)	3 (60)	n. s.*
ICI‑P	4 (44.4)	3 (50)	1 (33.3)	–
RI‑P	5 (55.6)	3 (50)	2 (66.7)	
Time from end of CCRT to Pneumonitis, months; median (IQR)	3 (2–6)	3 (1.75–7)	4 (2–5)	n. s.^#^
ICI‑P	7 (4.75–9.25)	7 (4.5–9)	8 (4.75–9.5)	–
RI‑P	2 (2–4)	2 (1–3)	3 (2–5.5)	
Deaths, *n* (%)	7 (17.9)	3 (10.3)	4 (40)	0.057^#^

* Fisher’s Exact test was applied

^#^ Mann-Whitney-U-Test was applied

*UICC* Union for International Cancer Control; *CTCAE* Common Terminology Criteria for Adverse Events, *ECOG* Eastern Cooperative Oncology Group, *SCC* Squamous cell carcinoma, *CCRT* Concurrent Chemoradiotherapy, *ICI‑P* Immune-checkpoint inhibitor induced pneumonitis, *RI‑P* Radiation-induced pneumonitis

All cases of pneumonitis occurred after the treatment with Durvalumab had already started. There was no significant difference observed between the two groups regarding of ICI discontinuation or reentry to ICI therapy. However, a significant difference was observed in the number of durvalumab cycles received by patients (*p* = < 0.007). The median time to discontinuation of Durvalumab was six months (range 1.5–11) in the 70 Gy group compared to two months (range 0.75–6) in the < 70 Gy group (*p* = 0.054). When comparing the time period of Durvalumab therapy, the 70 Gy group received a significantly longer duration of therapy compared to < 70 Gy group (*p* = 0.033).

One patient developed pneumonitis after discontinuing treatment due to liver toxicity. Overall, 15 (38.5%) patients developed pneumonitis, with ten patients (34.5%) in the 70 Gy dose group and five (50%) in the < 70 Gy total dose group. The response with pneumonitis was 1.9 times as large in the group with < 70 Gy compared to 70 Gy. Of all patients who developed pneumonitis, 26.7% had ICI‑P, while 73.3% had RI‑P. No significant difference in the incidence or type of pneumonitis was observed between the two groups or within the groups. Most patients (*n* = 14, 93.3%) developed CTCAE grade 2 (RI-P: *n* = 11; ICI-P: *n* = 3) and one patient developed CTCAE grade 3 pneumonitis (6.7%; ICI-P). Pneumonitis was further classified into acute toxicity (≤ 90 days after initiation of radiotherapy) and late toxicity (> 90 days after the initiation of radiotherapy). Acute toxicity was only seen for RI‑P, with 6 cases in total. (see Table 4).

### Tumor progression and overall survival

**Fig. 2:**
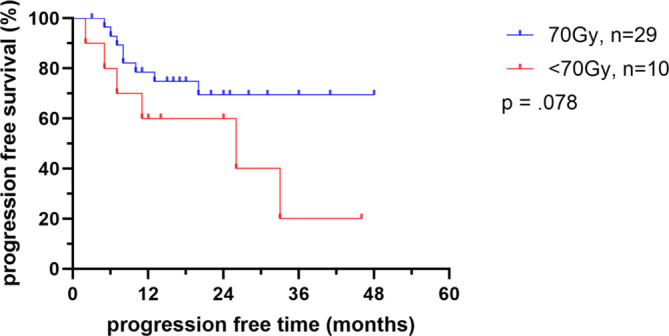
Tumor progression for each control group. Shown as Kaplan-Meier curve. Ticks marks indicate censored observations. Gy = Gray

Tumor progression occurred in 35.9% of all patients. In the 70 Gy total dose group, tumor progression was observed in 27.6%, while in the < 70 Gy group, it occurred in 60% of patients. The median time to tumor progression in both groups was approximately 8 months (range: 5.7–14.7) for those who progressed. No significant difference was found between the two groups in terms of tumor progression free survival time (Fig. 2).

Among patients who had tumor progression, 57.1% of cases occurred after discontinuation or completion of Durvalumab. In the 70 Gy group, 75% of patients had tumor progression after discontinuation or completion of Durvalumab, compared to 33.3% in the < 70 Gy group.

**Fig. 3:**
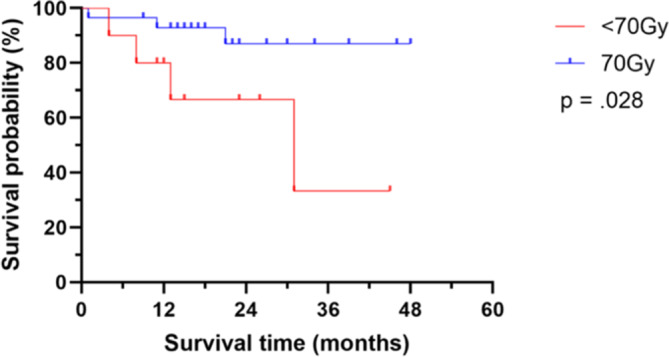
Overall survival time of patients receiving either 70 Gy or < 70 Gy total dose during concurrent chemoradiotherapy

In total, seven patients (four in the < 70 Gy group [40.0%] and three in the 70 Gy group [10.3%]) died within the analysis time range (Fig. 3 and Table 5). Median time to death was 11 months (range 4–21) for all patients that died. There was no significant difference in incidence of death between the groups, but there was a favorable trend towards the 70 Gy arm compared to the < 70 Gy group (*p* = 0.057). OS was significant different between the groups. Median OS was not reached in the 70 Gy group, while 6‑, 12-, 24-, 48- month OS rate were 96.6%, 93.1%, 89.7 and 89.7%, compared to the < 70 Gy group with a median OS of 31 months (*p* = 0.028). The 6‑, 12-, 24-, 48- month OS rates in the latter group were 90%, 80%, 70 and 60%, respectively.

**Table 5:** Overall survival

	All patients	70 Gy	< 70 Gy	*p* -value
*N*	39	29	10	–
*Death, n (%)*				
Yes	7 (17.9)	3 (10.3)	4 (40)	0.057°
No	32 (82.1)	26 (89.7)	6 (60)	–
Overall survival time (months; median, IQR)	21 (13–34)	21 (14.5–34)	14 (10.25–27.25)	n. s.°
Survival time (patients that died) (months; median, IQR)	11 (4–21)	11 (–)	10.5 (5–26.5)	–

° independent sample t‑test

On univariate analysis for OS, there was no difference between age groups, gender, histology, performance status, discontinuation of therapy, and pneumonitis occurrence.

#### Radiotherapy dose values

The MLD for the total lung in the 70 Gy group was 17.6 Gy (15.2–17.8) and 15.9 Gy (10.6–17.5) in the < 70 Gy group. A significant difference was found in MLD specifically in the ipsilateral lung, with 24 Gy (21.7–24.8) in the high dose group, and < 22.6 Gy (15.3–22.6) in the < 70 Gy group (*p* = 0.044).

**Fig. 4:**
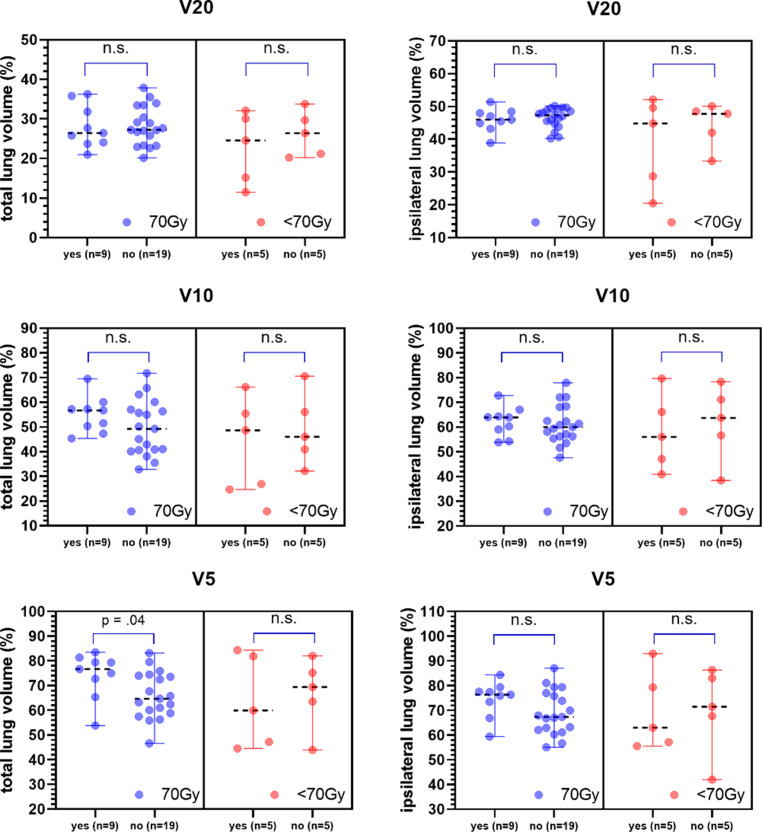
Differences in volume receiving doses (Vx) for patients with and without pneumonitis for both groups. Dots represent each patient. Median plus range is shown. Black dashed line marks median. *RI‑P* Radiation induced pneumonitis; *ICI‑P* Immune checkpoint inhibitor pneumonitis; *V20* Volume receiving doses above 20 Gy; *V10* Volume receiving doses above 10 Gy; *V5* Volume receiving doses above 5 Gy

When comparing the incidence of pneumonitis within the treatment groups, there was only one significant difference concerning the Vx-values. The total lung volume receiving more than 5 Gy (V5) was significantly higher in the 70 Gy group for those who got pneumonitis (*p* = 0.04). All Vx-values are shown in Fig. 4.

## Discussion

This is the first study evaluating the incidence of pneumonitis, either by radiotherapy or by ICI, in patients with NSCLC who are treated with CCRT, followed by Durvalumab in two different total radiation dose cohorts.

The results showed that 39% of patients developed any type or grade of pneumonitis, with 35% in the 70 Gy group (70% RI‑P; 30% ICI-P) compared to 50% in the < 70 Gy group (80% RI‑P; 20% ICI-P). These finding are consistent with previous literature, such as the PACIFIC trial by Antonia et al., which reported a pneumonitis rate of 34%, with 11% attributed to immune-related pneumonitis [1]. Similar results were reported by Shintani et al. [15]. In our study, most patients developed grade 2 pneumonitis, indicating a moderate level of severity, while less patients experienced grade 3 pneumonitis. Those findings are again in line with other authors [1, 16]. Furthermore, in both groups, the median time to onset of any cases of pneumonitis was similar (3 vs. 4 months), which is also comparable with the literature [17].

A main finding is that the OS was significantly different between patients receiving 70 and < 70 Gy (*p* = 0.028). In the < 70 Gy group median OS was 31 months, whereas in the 70 Gy group the median OS was not yet reached. This suggests that patients in the 70 Gy group had a better OS outcome compared to those in the less than 70 Gy group. This might indicate a potential benefit associated with a higher radiation dose, despite other studies reporting the opposite [3–6]. Moreover, the < 70 Gy group demonstrated a higher incidence of tumor progression (60%) compared to the 70 Gy group (27.6%). However, these findings may be seen as a potential confounder because patients with high mean lung doses, which imposed a high dose constraint, were administered less than 70 Gy total tumor dose, despite of a larger tumor mass. In other words, this is due to the fact that the dose is meant for the tumor but not the lungs, and with very large tumors it may happen that the individual tumor dose is lower, otherwise it would exceed dose constraint for the lung. Furthermore, patients receiving < 70 Gy maybe were considered more co-morbid, or with lower performance score. However, ECOG (Eastern Cooperative Oncology Group) status, age, gender, NSCLC disease stage, tumor histology, and tumor location were well balanced and did not differ between the groups.

OS data were still immature at the time of analysis, but the 70 Gy group showed promising results. After 12 months, the OS rate was 93.1%, and it remained at almost 90% over the next two years. These findings are comparable to other studies such as the “Five-Year Survival Outcome from the PACIFIC trial” by Spigel et al. [18], or other reports with an 12 months OS between 86 and 92% [2, 19]. Other studies that used higher radiation dose reported worse overall survival compared to those who received the standard dose of 60 Gy [6]. However, these studies did not used Durvalumab for consolidation therapy and applied up to 74 Gy.

In this study, 31% of the patients completed the recommended one year of Durvalumab treatment without discontinuation. No patient was still on maintenance therapy at the time of analysis. These findings are consistent with current literature, which report of 29 or 42% completion rates of the ICI consolidation therapy [20, 21]. The most common reasons for discontinuation of Durvalamub were mainly pneumonitis, or disease progression. The Society for Immunotherapy of Cancer recommends considering drug re-challenge in patients with grade 2 pneumonitis that has completely resolved, as well as in selected patients with grade 3 pneumonitis [22]. In this analysis, a third of the patients who experienced pneumonitis and required treatment, re-entered ICI treatment successfully. No relapse of pneumonitis was observed, and only one patient had tumor progression after re-entering. Furthermore, 75% of patients were able to continue treatment after re-entering. This finding highlights the potential benefits of considering ICI re-challenge more frequently [10, 20, 23–26].

The planning target volume and MLD are crucial factors in determining the optimal total radiation dose for patients undergoing concurrent chemoradiotherapy for unresectable NSCLC [4]. In this study, patients with high MLD which led to high dose constraint, received less than 70 Gy total prescribed dose. This suggests that the consideration of lung dose constraints, particularly the MLD, influenced the decision to limit the total radiation dose to ensure the safety of patients. The adherence to dose constraints aims to minimize the risk of radiation-induced lung toxicity, including pneumonitis. Interestingly, we observed a significant difference in mean dose in the ipsilateral lung. This difference can be attributed to adapting treatment planning strategies and the effort to achieve adequate target coverage while minimizing lung exposure. Furthermore, the mean dose for the total lung did not exceed the safety criterion of MLD ≤ 20 Gy for both groups [27]. This indicates that, despite differences in mean dose between the groups, radiation doses to the entire lung were kept within acceptable limits to reduce the risk of radiation-induced lung toxicity. There were no significant differences in V20, V10 and V5 between the groups. However, total lung volume receiving more than 5 Gy (V5) was significantly higher in the 70 Gy group among patients with radiation-induced pneumonitis. Radiotherapy guidelines for NSCLC suggest V5 should be kept below 60% to minimize the risk of pneumonitis [28]. The V5 values in the 70 Gy group with RI‑P in this study exceeded the recommended threshold, which might has contributed to the appearance of a RI‑P. These results highlight the importance of considering not only the MLD but also specific dose-volume parameters in assessing the risk of pneumonitis.

### Limitations

An important limitation of this study is the very small sample size. Being conceptualized as a retrospective pilot study, the group size is especially low for patients receiving < 70 Gy total radiation dose. The study focuses on the appearance of pneumonitis, and the higher radiation dose was not inferior in this matter. Other evaluations, such as survival, must be interpreted very cautiously since the study was not powered accordingly. On the other side, this study provides interesting information and the data is new. Future prospective studies should be planned with sample size calculation and a possible randomization.

Since this was a retrospective design, randomization was not possible. Another issue in this context is that tumor size is considered a potential confounding factor, because radiation dose of the control group with < 70 Gy mainly depended on MLD, V20, V10, and V5 values. Randomization in future studies would reduce this possible limitation.

Another limitation is a potential misclassification bias in differentiating between ICI‑P and RI‑P, as the information regarding pneumonitis may be concealed within the radiation field.

There is an ongoing discourse on the probability of bias creation following the administration of antiangiogenic drugs due to the presence of antiangiogenetic food components. In this study, no data on dietary behavior was collected. Future studies should use a validated semi-quantitative food frequency questionnaire to gather information on these agents. [29].

Lastly, the inclusion criterion for the study was based on potentially completing one year of Durvalumab therapy. However, for survival analysis, the total duration of therapy since its initiation was collected, which varied for each patient. This difference in data collection may introduce bias due to the varying observation periods.

## Conclusions

In summary, CCRT with a total radiation dose of 70 Gy followed by consolidation therapy with Durvalumab appears to be a safe treatment regimen, that did not result in higher pneumonitis rates compared to the standard protocol in our study cohort.

The results suggest that the use of a 70 Gy total radiation dose for CCRT followed by immunotherapy with Durvalumab may offer potential clinical benefits. The higher radiation dose appears to be associated with improved OS outcomes. While the incidence of pneumonitis observed is consistent with previous findings, cases of pneumonitis remain a potential factor for treatment discontinuation. Re-entry to ICI therapy should be carefully evaluated and considered. Further research is necessary to better understand the risk factors and to develop optimal management strategies for pneumonitis in this specific patient population, particularly when distinguishing between RI‑P and ICI‑P. Furthermore, the findings highlight the importance of careful treatment planning to minimize lung toxicity, particularly in terms of MLD, and V5 values. Prospective studies with larger sample sizes and longer follow-up periods are warranted to validate these results.

## Data Availability

The datasets used and analyzed during the current study are available from the corresponding author on reasonable request.

## Abbreviations

CCRT: Concurrent chemoradiotherapy
CTCAE: Common Terminology Criteria for Adverse Events
ICI: Immune checkpoint inhibitor
ICI‑P: Immune checkpoint inhibitor pneumonitis
MLD: Mean lung dose
NSCLC: Non small-cell lung cancer
OS: Overall survival
PD-L1: Programmed death ligand 1
PFS: Progression free survival
RI‑P: Radiation pneumonitis
RT: Radiotherapy
SCC: Squamous cell carcinoma
UICC: Union of International Cancer Control
